# Bifurcation Theory Approach to Neuro-Developmental Language Impairment in Autistic Children

**DOI:** 10.21315/mjms2018.25.4.14

**Published:** 2018-08-30

**Authors:** Somayeh Sadat Hashemi Kamangar, Fatemeh Bakouie, Shahriar Gharibzadeh

**Affiliations:** 1 Institute for Cognitive and Brain Sciences, Shahid Beheshti University, Tehran, Iran; 2 Daneshjou Boulevard, District 1, Tehran, Iran; 3 Basir Eye Health Research Center, Tehran, Iran

**Keywords:** dynamical system theory, language impairment, autism

## Abstract

Autism Spectrum Disorders (ASD) is defined by a spectrum of deficits in social interactions and limited or stereotyped patterns of behaviours. Consequently, language as the result and the tool of communication is impaired in individuals affected by ASD. Thus, a better knowledge of language impairment leads to a better solution of the communicating problems. “Dynamical systems theory” could help us understand the development of language and the related impairments. Based on this theory, language development can be considered as a dynamical system with the trajectory of variation. In the present study, we hypothesised that a kind of bifurcation happens in language development trajectory of autistic children when they reach a higher level of language like pragmatics.

Autism Spectrum Disorders (ASD) is neurodevelopmental deficit characterised by serious impairments in the domain of communication and social interaction (1). Consequently, language is impaired among individuals affected by ASD as it is regarded as the result and tool of communication. Further, language impairment should be identified and treated although it is not the core symptom of ASD (1). Furthermore, language impairment as the symptom of ASD has a spectrum in which there are autistic patients with no language impairment in one extreme (1) and some with impairments in structural language abilities such as phonology, syntax and semantics in another extreme. In the middle of these two extremes, there are the autistic individuals with no language impairment except in pragmatics which is related to the context (1). In addition, they have some difficulties in understanding some expressions such as irony or metaphors, and proverbial phrases (2). Further, most of the autistic individuals have language impairments in pragmatics (1, 2). The present study aims to focus on these majorities. It is worth noting that pragmatic language impairment is the autistic children’s feature due to their social interaction difficulties. In general, developed children may show individual differences in language acquisition, but all of them will finally comprehend pragmatics which is the highest level of language development. However, autistic children have problems in processing those levels of language which is related to the contexts such as metaphor and irony which needs social interaction for understanding. Human language passes different language levels during developmental process, from birth to late childhood and adolescence. Language skills change through time from the “lowest level” to the “highest” one, called “pragmatics”. Further, language develops from the representation of informative actions and communicative intentions to lexicon/ semantic processing, syntactic analysis and pragmatic integration (3). Currently, the focus on theoretical explanation and finding the nature of the basic developmental mechanisms comes mainly from a “system-oriented” research in which the theory of nonlinear complex dynamical systems is used (4). According to this theory, “dynamical systems theory” can help us understand the developmental process of language and the related impairments (5). In other words, the temporal development of language can be studied by Nonlinear Dynamic Models (NDM). NDMs can perfectly model the complex processes such as development, where the change of the output is not proportional to the change of the input (4, 6).

Dynamical systems theory deals with the long-term qualitative behaviour of dynamical systems (7). A dynamical system consists of a set of variables which describe its state and a law related to the evolution of the state variables with time (8). In summary, dynamical systems theory is regarded as a tool for describing how one state develops into another, over the course of time (8). Thus, language development could be considered as a dynamical system with the trajectory of variation. According to dynamical systems theory, a change in the controlling parameters results in changing the qualitative structure of the trajectories. For instance, the fixed points in which the derivative of the variables is zero can be created or destroyed, or their stability can change. These qualitative changes in dynamics are called “bifurcation”, and the parameter values in which they occur are named “bifurcation points”. In addition, the qualitative changes are considered as a bifurcation while they are great, compared to the small changes of the system and long-term, compared to the short-term changes of the system (7).

The present study aimed to propose a bifurcation theory approach for language impairment in ASD. In this regard, the following hypotheses were raised:

H1: A kind of bifurcation happens in language development trajectory of autistic children. In fact, a considerable change occurs in the qualitative structure of the trajectory.
  - In the beginning, the trajectory is related to the “lower level” processing of language like lexicon/semantic processing.
  - As the trajectory evolves, “higher level” aspects of language like syntax and pragmatics will be processed. Therefore, autistic individuals with normal language have the same trajectory of language development with those having pragmatic impairment until they reach a critical point in which the bifurcation happens.
H2: At the bifurcation point, the same path is separated into two different paths including:
  - A normal trajectory which approaches the highest level of language development or pragmatic integration, and
  - A trajectory with inappropriate variations leading to the misunderstanding of the pragmatics.

At the bifurcation point, the initial values of the system determine the future path of the system, which is related to the normal or impaired state of the language. Therefore, the parameters of language development system such as individuals’ age, environmental and social conditions, and genetics can determine the final state of language trajectory, among which genetics plays the greatest role. Based on the literature, there are some genome-wide genetic links between ASD and typical variation in social behaviour (9). In addition, mutations could result in distorting typical neuronal connectivity, increasing the risk of ASD, and creating the difficulties in adapting to external stimuli such as those received during social interactions (10). These may be related to some main reasons why pragmatic impairment, which refers to the context and social interaction, occurs in autistic children. Therefore, genetics is considered as an influential control parameter, leading to a bifurcation in language development trajectory. Time series design based on the children's linguistic data is necessary to use this approach for studying language development. Regarding NDMs, many samples are necessary to get an adequate adjustment. Further, the study should cover a wide range of ages for the children to examine all aspects of language development from the lower level of processing to high levels of syntax and pragmatics. It is worth noting that we should have the certitude due to autism, not to any other circumstances when a deviation from the normal pattern is detected in a subject. Therefore, the autistic and control group should be selected carefully in the experimental design.

Bifurcation theory was already proposed in neuroscience, cognitive science, and psychology. For example, this theory was used in the dynamical model of neuronal activities, as well as modeling cognitive functions like visual perceptions or sudden changes in an individual’s cognitions and behaviours (11, 12, 13). In the present study, dynamical systems approach was proposed for modeling language development by using bifurcation theory, which may help us to model this developmental problem in the language of individuals with autism. By finding and tuning the bifurcation parameter, the problem may be solved. Further, predicting, preventing and treating language impairment among the individuals with autism may be achieved by controlling the bifurcation parameters and identifying the desired initial values of language development system.
